# Body Composition Predictors of Adverse Postoperative Events in Patients Undergoing Surgery for Long Bone Metastases

**DOI:** 10.5435/JAAOSGlobal-D-22-00001

**Published:** 2022-03-09

**Authors:** Peter K. Twining, Olivier Q. Groot, Colleen G. Buckless, Neal D. Kapoor, Michiel E. R. Bongers, Stein J. Janssen, Joseph H. Schwab, Martin Torriani, Miriam A. Bredella

**Affiliations:** From the Department of Orthopaedic Surgery–Orthopaedic Oncology Service, Massachusetts General Hospital–Harvard Medical School, Boston, MA (Mr. Twining, Dr. Groot, Dr. Kapoor, Dr. Bongers, Dr. Schwab); the Division of Musculoskeletal Imaging and Intervention, Department of Radiology, Massachusetts General Hospital and Harvard Medical School, Boston, MA (Dr. Buckless, Dr. Torriani, Ms. Bredella); and the Department of Orthopedic Surgery, Amsterdam Movement Sciences, Amsterdam University Medical Center–University of Amsterdam, Amsterdam, the Netherlands (Janssen).

## Abstract

Body composition assessed using opportunistic CT has been recently identified as a predictor of outcome in patients with cancer. The purpose of this study was to determine whether the cross-sectional area (CSA) and the attenuation of abdominal subcutaneous adipose tissue, visceral adipose tissue (VAT), and paraspinous and abdominal muscles are the predictors of length of hospital stay, 30-day postoperative complications, and revision surgery in patients treated for long bone metastases.

**Methods:**

A retrospective database of patients who underwent surgery for long bone metastases from 1999 to 2017 was used to identify 212 patients who underwent preoperative abdominal CT. CSA and attenuation measurements for subcutaneous adipose tissue, VAT, and muscles were taken at the level of L4 with the aid of an in-house segmentation algorithm. Bivariate and multivariate linear and logistic regression models were created to determine associations between body composition measurements and outcomes while controlling for confounders, including primary tumor, metastasis location, and preoperative albumin.

**Results:**

On multivariate analysis, increased VAT CSA {regression coefficient (r) (95% confidence interval [CI]); 0.01 (0.01 to 0.02); *P* < 0.01} and decreased muscle attenuation (r [95% CI] −0.07 [−0.14 to −0.01]; *P* = 0.04) were associated with an increased length of hospital stay. In bivariate analysis, increased muscle CSA was associated with increased chance of revision surgery (odds ratio [95% CI]; 1.02 [1.01 to 1.03]; *P* = 0.04). No body composition measurements were associated with postoperative complications within 30 days.

**Discussion:**

Body composition measurements assessed using opportunistic CT predict adverse postoperative outcomes in patients operated for long bone metastases.

Treatments for neoplastic disease have rapidly improved over the past decades, and many patients are surviving longer, resulting in an increased likelihood of bone metastases.^1^ In patients with prolonged expected survival, surgical management is often considered for extremity bone metastases to improve quality of life and protect against impending pathologic fractures.^1^ Other treatment strategies of bone metastases include radiation therapy or chemotherapy. Because surgery is not without complications, risks and benefits of various treatment options must be thoroughly explored. Many prognostic tools, from simple scoring systems to machine learning algorithms for predicting mortality after surgical management of metastatic bone disease, have been developed to aid surgeons in this decision-making process.^2,3,4,5,6,7,8,9^ However, it is also important to consider the possible consequences of surgical management, such as prolonged hospital stays, postoperative complications, and revision surgeries.^10,11^ There is a paucity of literature on establishing risk factors for these outcome measures.

Assessment of body composition using CT done for other purposes, so-called opportunistic CTs, is able to predict outcome in patients with cancer.^12-14^ The most common CT body composition measurements used are attenuation and cross-sectional area (CSA) of abdominal subcutaneous adipose tissue (SAT), visceral adipose tissue (VAT), and paraspinous/abdominal muscle. Recent studies have shown some of these CT body composition measurements to be associated with increased length of hospital stay (LOS), readmission, postoperative complications, and other adverse outcomes in patients with various gastrointestinal malignancies.^15,16,17,18^ However, the association of these measurements with adverse postoperative events in patients with long bone metastases undergoing surgery remains unexplored.

The purpose of this study was to determine whether CSA and attenuation of abdominal SAT, VAT, and paraspinous and abdominal muscles obtained using opportunistic CT can predict LOS, 30-day postoperative complications, and revision surgery in patients undergoing surgery for long bone metastases.

## Patients and Methods

### Patients and Study Design

A retrospective database of patients who underwent surgery for long bone metastases at a single tertiary care center from January 1, 1999, to December 31, 2017, was used for this study.^2^ Inclusion criteria included (1) patients older than 18 years, (2) surgically treated for long bone metastatic disease (including lymphoma and multiple myeloma),^6^ and (3) a preoperative CT scan within 3 months before the operation. Exclusion criteria were (1) multiple metastatic bone tumors requiring surgery; (2) revision surgeries; (3) surgery type other than intramedullary nailing, endoprosthetic reconstruction, plate and screw fixation, dynamic hip screw, or any combination thereof; or (4) a CT scan unusable because of poor quality or no inclusion of the fourth lumbar vertebrae (L4) level.

Surgical decision-making for prophylactic fixation in these patients was based on shared decision-making guided by the Mirels^19^ score. For patients who underwent multiple abdominal CT scans before surgery, the scan closest to the date of operation was chosen. Similarly, for patients who underwent multiple operations for long bone metastases, only the first operation was included.

### CT Analysis

Preoperative abdominal CTs were used for analysis. CSA and attenuation measurements of VAT, SAT, and muscles were determined at the level of L4. For CSA measurements, all scans were used. For attenuation measurements, only noncontrast scans were used. CT devices, protocol, and analysis methods have been described previously.^20^ Briefly, scans at the L4 level were analyzed by an automated in-house algorithm and adjusted by a trained researcher (C.G.B.) under the supervision of senior fellowship-trained musculoskeletal radiologists (M.T. and M.A.B.).

### Variables and Outcomes

Outcome variables were (1) LOS (days), (2) postoperative complications within 30 days, and (3) revision surgery. Postoperative complications included pneumonia, venous thromboembolism, sepsis, myocardial infarction, wound infection and/or dehiscence, and urinary tract infection.^21^ In addition to CT body composition measurements, variables believed to be associated with postoperative complications were collected from the electronic medical records. These included age, sex, body mass index (BMI), duration from primary diagnosis until operation (days), Charlson Comorbidity Index,^22^ preoperative albumin (g/dL), race, primary tumor growth category according to Katagiri et al,^5^ additional metastases outside the lesion being treated for, location of bony metastasis (upper or lower extremity), type of surgery, previous radiation therapy, previous systemic therapy, and presence of a pathological fracture.

### Statistical Analysis

Bivariate analysis was used to assess the associations of explanatory variables with all three outcomes. Linear regression was used for continuous outcomes (LOS) and logistic regression for categorical outcomes (complications within 30 days and revision surgeries). All clinical variables with a *P* value less than 0.10 in bivariate analysis were included in multivariate analysis. Collinearity was tested before performing multivariate analyses, and BMI was excluded because of high collinearity with the body composition measurements. Body composition measurements with *P* < 0.10 were included separately in the multivariate analyses. Multiple imputations were applied to estimate missing values for BMI in eight patients (3.8%) and albumin in 5 patients (2.4%). No multiple imputation was done for the missing attenuation measurements because this was the explanatory variable of interest. No sample size was calculated because all eligible patients between 1999 and 2017 were included. For all analyses, a two-sided *P* value of <0.05 was considered significant. All statistical analyses were done using Stata 15.0 (StataCorp LP), R version 3.6.3 (The R Foundation, Vienna, Austria), and R Studio version 1.3.887 (RStudio).

## Results

### Patients and Characteristics

**Figure 1:**
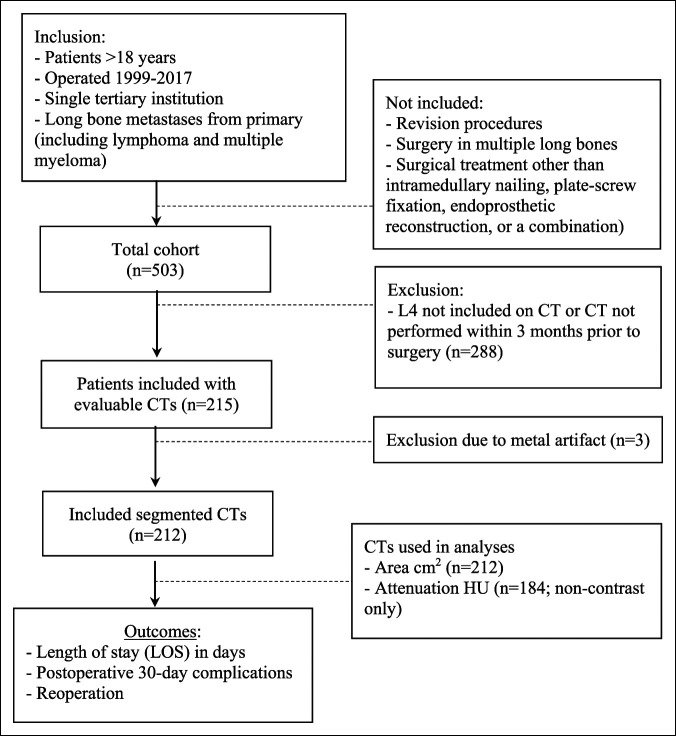
Flow diagram depicting the patient selection.

Of the 503 patients identified who underwent surgery for long bone metastases, 212 had CT scans and met the inclusion criteria (Figure 1.) Only CT scans without intravenous contrast (n = 184) were used for attenuation measurements. Baseline patient characteristics are provided in Supplemental Table 1, http://links.lww.com/JG9/A196. The median age of study participants was 63 years (interquartile range [IQR] 56 to 69), with 49% being male and 51% female. Seventy-six percent of the patients were treated for lower extremity and 24% for upper extremity bone metastases. The most common primary tumor types were lung (22%), renal (15%), and breast (15%) (Supplemental Table 2, http://links.lww.com/JG9/A197). Ninety-day mortality was 32%, and 1-year mortality was 64%. The median LOS was 5 days (IQR 5 to 7); 10% (21 patients) experienced postoperative complications within 30 days and 4.7% (10 patients) had a revision surgery.

Patients included in the study group had a higher Charlson comorbidity score, higher proportion of primary tumors in the moderate and rapid tumor growth categories, were more likely to have additional metastases besides the surgically treated lesion and had a longer LOS than the group excluded because of inadequate or absent CT scans (Supplemental Table 3, http://links.lww.com/JG9/A198).

### Length of Stay

On bivariate analysis, increased VAT CSA {regression coefficient (r) (95% confidence interval [CI]) = 0.01 (0.01 to 0.02), *P* = 0.01} and decreased muscle attenuation (r [95% CI] = −0.08 [−0.15 to −0.01], *P* = 0.03) were associated with longer LOS (Supplemental Table 4, http://links.lww.com/JG9/A199). Three clinical variables were controlled in multivariate analysis: preoperative albumin level, primary tumor growth, and metastasis location. On multivariate analysis, increased VAT CSA (r [95% CI] = 0.01 [0.01 to 0.02], *P* < 0.01) and decreased muscle attenuation (r [95% CI] = −0.07 [−0.14 to −0.01], *P* = 0.04) were associated with longer LOS after controlling for all three clinical variables (Tables 1 and 2).

**Table 1:** Multivariable Logistic Regression Analysis for VAT Area and Length of Stay After Surgery for Long Bone Metastases Using Pooled Imputed Data

Variables	Coefficient (95% CI)	Standard-error	*P*
Preoperative albumin (g/dL)	−1.95 (−3.04 to −0.86)	0.555	**<0.01**
Primary tumor growth			
Slow	0.71 (−0.92 to 2.34)	0.827	0.39
Moderate	2.13 (0.51 to 3.75)	0.823	**0.01**
Rapid	Reference value		
Metastasis location			
Upper extremity	−1.90 (−3.49 to −0.31)	0.808	**0.02**
Lower extremity	R value		
VAT area (cm^2^)	0.01 (0.01 to 0.02)	0.004	**<0.01**

CI = confidence interval, VAT = visceral adipose tissue

Bold *P* values are <0.05.

**Table 2:** Multivariable Logistic Regression Analysis for Muscle Attenuation and Length of Stay After Surgery for Long Bone Metastases Using Pooled Imputed Data

Variables	Coefficient (95% CI)	Standard error	*P*
Preoperative albumin (g/dL)	−1.74 (−3.02 to −0.46)	0.648	**0.01**
Primary tumor growth			
Slow	0.44 (−1.43 to 2.31)	0.949	0.65
Moderate	2.40 (0.55 to 4.24)	0.935	**0.01**
Rapid	Reference value		
Metastasis location			
Upper extremity	−1.47 (−3.32 to 0.38)	0.935	0.12
Lower extremity	R value		
Muscle attenuation (HU)	−0.07 (−0.14 to −0.01)	0.034	**0.04**

CI = confidence interval, HU = Hounsfield Units

Bold *P* values are <0.05.

### Postoperative Complications Within 30 Days

On bivariate and multivariate analysis, no explanatory variables or body composition measurements were associated with postoperative complications within 30 days (Supplemental Table 4, http://links.lww.com/JG9/A199).

### Revision Surgery

On bivariate analysis, increased muscle CSA was associated with an increased likelihood of revision surgery (odds ratio [95% CI]; 0.02 [0.01 to 0.03]; *P* = 0.04, Supplemental Table 4, http://links.lww.com/JG9/A199). Multivariate analyses were not controlled for confounders because no clinical variables had *P* values below 0.10. No other body composition measurements were associated with revision surgery (*P* > 0.05). Patients who underwent a revision surgery had a longer postoperative follow-up as compared with patients who did not undergo a revision surgery (mean follow-up in months: revision surgery 20 months versus no revision surgery 14 months). Patients who had higher muscle mass had longer survival (median [IQR]; 261 [77 to 691]) as compared with patients who had lower muscle mass (median [IQR]; 98 [46 to 434]).

## Discussion

Assessment of body composition measurements on readily available preoperative CT scans in patients with cancer could provide prognostic information for survival and adverse postoperative outcomes. Body composition measurements from these opportunistic CT scans are associated with adverse postoperative outcomes in various patient populations.^15,16,17,18^ However, to the best of our knowledge, this study is the first study to explore the effects of area and attenuation of SAT, VAT, and muscles on LOS, postoperative complications, and revision surgeries in patients operated for long bone metastases. Our study shows that (1) increased VAT area and decreased muscle attenuation are associated with longer LOS while controlling for several covariates and (2) increased muscle area is associated with increased chances of revision surgery in patients surgically treated for long bone metastases. No association was found between body composition measurements and postoperative complications. This work expands on the growing body of literature that body composition assessed by opportunistic, preoperative CT may be useful for prognostication in patients with metastatic disease.

### Visceral Adipose Tissue Area

Our findings that an increased VAT area is associated with an extended LOS are consistent with previous studies on different disease types in several patient populations.^15,16,18,23,24^ Two recent studies of 139 and 110 patients have shown that an increased VAT area was associated with increased postoperative complications in patients who underwent surgery for gastric or colorectal cancer.^18,24^ Although our study did not show a relationship between VAT area and postoperative complications, both increased LOS and complications are adverse outcomes that likely result from poor overall health. In a study of 2,100 patients, increased VAT area was shown to be associated with increased risk for readmission after surgery for colorectal cancer.^16^ In addition, patients undergoing surgery for diverticular disease showed an association between increased VAT area and increased postoperative complications.^15^ It has been proposed that the adverse effects of visceral adiposity on outcome may be due to its effect on cardiometabolic risk, including higher incidence of hypertension, diabetes, and the metabolic syndrome.^25,26^ In patients with colorectal cancer, VAT is superior to BMI in predicting the presence of cardiometabolic comorbidities.^27^ The consistency of the association of VAT with adverse effects across different disease types and surgical locations supports increased VAT area as a marker of poor overall health.

### Muscle Attenuation

We found that decreased muscle attenuation was associated with longer LOS. This is consistent with other studies in different patient populations showing poor outcomes associated with decreased muscle attenuation.^13,28,29^ In a study of 805 patients after surgery for colorectal cancer, decreased muscle attenuation was associated with longer LOS.^30^ Decreased muscle attenuation is reflective of intramuscular fat deposition, known as myosteatosis, which is associated with cancer cachexia.^31,32^ In addition, myosteatosis, both in isolation and when combined with visceral obesity, is associated with longer LOS in an international cohort of 2,100 patients who underwent surgery for colorectal cancer.^16^ Future studies could explore how different combinations of body composition factors may contribute to outcomes. Changes in the composition of certain body tissues likely do not occur in isolation because there is complex cross talk between adipose tissue and muscle in patients with cancer cachexia.^13,33,34^

### Muscle Area

Our finding that increased muscle area was associated with increased revision surgeries is an unexpected finding. A systematic review on patients undergoing abdominal surgery found that low, not high, muscle area was a risk factor for postsurgical adverse events.^35^ Another systematic review found sarcopenia, defined as low muscle area, was associated with increased mortality and postoperative complications in surgical oncology patients.^36^ This finding seems paradoxical in that an increased muscle area would be associated with revision surgery because increased muscle mass is generally present in patients with better overall health status. However, it may be that increased revision surgeries are a consequence of the prolonged survival in this group (additional analysis not shown). Patients with metastatic disease likely have a higher chance of additional operations if they are living longer.

### Implications for Practice

Several prognostic models have been developed to assess survival in patients with metastatic bone disease.^2,37,38^ To develop scoring systems and algorithms, easily identifiable and interpretable variables associated with those outcomes must be identified. Patients with metastatic bone lesions generally already have preoperative CT scans available, so these so-called opportunistic CT scans can be used to assess body composition measurements which can be incorporated into prediction models. Our results show that an increased VAT area and decreased muscle attenuation are both associated with increased length of stay. We do not advocate for the use of these metrics to deny patients' surgery. Instead, future prediction models should take into account multiple aspects, including survival and risks of adverse outcomes, such as complications, length of stay, revision surgeries, and potential quality of life benefits, to provide surgeons and patients with robust information on which to guide their clinical decisions. In addition, tools that aid in determining a patient's length of stay would be especially useful for discharge planning as well as on a systems-level for hospital bed availability. We believe that the CT-defined body compositions measures of VAT area and muscle attenuation presented in this study will be a helpful predictive tool in this prognostication process.

### Limitations

This study has several limitations. First, this was a retrospective study and should be interpreted in the appropriate context. To strengthen our retrospective design, CT measurements were made by a researcher blinded to outcomes to mitigate potential observer bias. Second, despite the large cohort size, during our patient selection process, over 50% of our originally identified cohort was excluded from the analysis because of inadequate or unavailable CT scans. A baseline comparison between the included and excluded groups showed that the included group had a higher Charlson comorbidity score, a higher proportion of rapidly growing tumor types were more likely to have additional metastatic lesions, and a longer LOS. These differences suggest that the included group sustained more advanced diseases and comorbidities than the excluded group. It is reasonable that the more fragile group would be more likely to have preoperative CT scans for cancer staging and surveillance. It is also possible that the results found in this study would not extrapolate to the excluded group. Future studies across various populations would be required to assess the generalizability of these findings. These studies should prospectively include CT-defined body composition measurements to evaluate the prognostic value for these variables' adverse postoperative outcomes. In addition, improving or maintaining quality of life is recognized as an important outcome to prioritize when evaluating a patient for surgical management of long bone metastases and must be considered alongside survival benefits and risk of complications. Despite these limitations, our large cohort size, in addition to controlling for several known confounding variables, lends validity to this study.

In conclusion, body composition measurements, assessed using opportunistic CT, predict adverse postoperative outcomes in patients operated for long bone metastases. These measures could be incorporated into existing prognostic models to aid physicians and patients in clinical decision-making.
